# NOVEL INSIGHTS IN ADVANCED THYROID CARCINOMA: FROM MECHANISMS TO TREATMENTS: Molecular insights into the origin, biology, and treatment of anaplastic thyroid carcinoma

**DOI:** 10.1530/ETJ-25-0057

**Published:** 2025-06-02

**Authors:** Amir Hossein Karimi, Peter YF Zeng, Matthew Cecchini, John W Barrett, Harrison Pan, Shengjie Ying, Nhi Le, Joe S Mymryk, Laurie E Ailles, Anthony C Nichols

**Affiliations:** ^1^ Department of Otolaryngology – Head & Neck Surgery, Western University, London, Ontario, Canada; ^2^ Department of Pathology and Laboratory Medicine, Western University, London, Ontario, Canada; ^3^ Department of Oncology, Western University, London, Ontario, Canada; ^4^ Department of Microbiology & Immunology, Western University, London, Ontario, Canada; ^5^ Princess Margaret Cancer Centre, University Health Network, Toronto, Ontario, Canada

**Keywords:** anaplastic thyroid carcinoma, genomics, targeted therapy, immunotherapy

## Abstract

Anaplastic thyroid carcinoma (ATC) is among the most daunting entities in clinical oncology. Large-scale genomic studies of thyroid cancer within the last decade have uncovered a distinct set of recurrent somatic alterations implicated in the development, aggressiveness, and treatment resistance of ATC. The sequence of events leading to the development of ATC commonly begins with a tumorigenic mutation that constitutively activates the mitogen-activated protein kinase (MAPK) pathway, giving rise to indolent entities such as well-differentiated papillary or follicular thyroid carcinomas. This is followed by recurring alterations that drive oncogenic properties such as enhanced proliferation, genomic instability, replicative immortality, and dedifferentiation, culminating in the emergence of highly aggressive ATC tumors. The truncal MAPK-activating events present therapeutic opportunities, as small molecule inhibitors against key components of this pathway are available. Indeed, genotype-guided targeting of the MAPK pathway is now the standard of care for subgroups of ATC patients, and further efforts exploring additional MAPK inhibitors and the combination of immune checkpoint blockade with MAPK inhibition are overcoming resistance to the current targeted therapies in the clinic and expanding our arsenal against this disease. In this review, we summarize the current understanding of the genomic landscape of ATC, discuss the biological and clinical ramifications of recurring aberrations, and provide an overview of the opportunities and challenges in the clinical management of this lethal malignancy.

## Introduction

Anaplastic thyroid carcinoma (ATC) is one of the most lethal and clinically challenging human malignancies. Patients with ATC often present with explosive tumor growth and extensive local invasion leading to airway and esophageal obstruction (1). Effective clinical management of ATC is hindered by its poor response rates to radioactive iodine, radiation, and chemotherapy. Complete or near-complete surgical resection of the tumor, followed by radiotherapy with or without chemotherapy, represents the standard of care for ATC patients with locoregionally confined resectable tumors and may prolong survival in carefully selected cases (1). However, this is rarely feasible, given the frequent invasion of the trachea, esophagus, and carotid sheath by the nonmetastatic tumors and the presence of distant metastases at diagnosis in half of the patients (2). Consequently, there is a striking difference in patient outcomes between ATC and well-differentiated thyroid cancer (WDTC, including papillary and follicular thyroid cancer): while WDTC tumors are some of the most indolent malignancies, so much so that overtreatment of these tumors is a cause for concern (3), ATC has a historical median overall survival of less than 6 months (4, 5).

However, the last decade has witnessed substantial improvements in the survival rates of ATC patients, with an impressive 50% reduction in the hazard of death at any given time when comparing patients treated between 2017–2019 and 2000–2013 (6). Moreover, a recent clinical trial reported an unprecedented median overall survival of 43 months in a subset of cases (7). These improvements are largely due to investigational efforts uncovering the molecular underpinnings of ATC, enabling the implementation of targeted therapies and informing the continued refinement of the standard of care.

Recently, the results of the global ATC initiative (GATCI), spearheaded by our team, have been published (8). Through this large-scale effort spanning 15 centers, the genomic profiles of 329 thyroid cancer regions were characterized with multiple platforms, further contributing to the body of research over the past decade that has advanced our understanding of aggressive thyroid tumors (8, 9, 10, 11, 12, 13, 14, 15, 16, 17, 18, 19, 20, 21, 22, 23, 24). Herein, we provide an overview of the current insights into the molecular landscape of ATC. We review the primary genomic dysregulations in ATC, discuss their biological and clinical implications, and offer an outline of the major frontiers in the clinical management of this aggressive disease.

## Moderately complex yet clinically devastating: the genomic landscape of ATC

**Figure 1:**
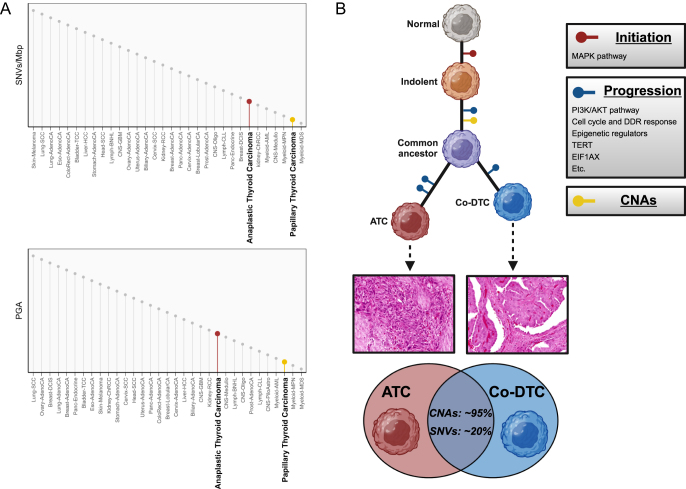
Moderate alteration burden and the stepwise evolution of ATC. (A) The comparison of the genomic alteration burden of tumors shows that, while ATC tumors exhibit higher alteration burdens compared to papillary thyroid cancer, they occupy a low-to-moderate position across the different cancer types (the lollipops solely show the increasing order of the alteration burdens of different cancer types and do not represent actual values). (B) A sequential acquisition of alterations drives the transformation of thyrocytes to ATC. This process is commonly initiated with an alteration constitutively activating the MAPK pathway, giving rise to premalignant or malignant entities with high levels of differentiation and indolent characteristics. This is followed by additional alterations that lead to further dedifferentiation of cells and the acquisition of aggressive traits. Consistent with this model, genomic features of well-differentiated thyroid tumors are also observed in the advanced tumors, reflecting their origin as a truncal event. In contrast, alterations driving the progression of the disease are rare and subclonal in well-differentiated tumors and become enriched in the advanced disease. Moreover, a co-occurring differentiated thyroid cancer (co-DTC) is frequently observed in ATC, consistent with a branching evolution during the development of the disease. The clonal separation of these two components can hypothetically occur at any stage of the disease progression. Representative high-power field images show ATC and co-DTC components of the same tumor. Genomic characterization of the co-occurring DTCs in the GATCI cohort indicated that co-DTC tracks with the advanced forms of thyroid cancer, and the common ancestor harbored ∼95% of CNAs and ∼20% of SNVs in the two components. The lengths of the lines in the depicted phylogenetic tree are hypothetical, as sufficient data to accurately estimate the average evolutionary distance of the different components is not available. The data and plots for this figure are adapted from (8). Mbp, megabase pair; PGA, percentage of genome altered; DDR, DNA damage response.

Despite its aggressive characteristics and lethality, the genomic complexity of ATC remains modest. While it has a higher single nucleotide variant (SNV) and copy number alteration (CNA) burden compared to WDTC (8, 9, 13), ATC on average harbors 3.8 SNVs/Mb and 120 CNAs and occupies a low-to-moderate position on the spectrum of genomic alteration burden across different cancer types (Fig. 1A) (8). Moreover, the majority of ATC tumors harbor highly recurrent alterations (8, 9, 10, 11, 12, 13), further reducing the complexity of the genomic landscape of ATC. The relatively low genetic complexity of ATC streamlines the identification of oncogenic alterations driving the disease. This also bears clinical implications, as it facilitates the development of prognostic/predictive patient stratification criteria, supports rapid testing at the time of diagnosis, and warrants concentrated efforts to develop effective precision interventions with widespread clinical applicability.

Molecular evidence in thyroid cancer to date strongly supports a model where a stepwise acquisition of alterations drives the progression of the disease, advancing from highly indolent WDTCs to extremely aggressive ATC tumors (Fig. 1B) (25). This malignant transformation of thyrocytes is primarily initiated by the constitutive activation of the mitogen-activated protein kinase (MAPK) pathway, with additional recurring alterations such as loss of p53 and activation of TERT and the PI3K/AKT pathway promoting more aggressive and less differentiated thyroid cancer. Supporting this model, genomic studies consistently indicate that the advanced forms of thyroid cancer share the genomic features of WDTC but are also enriched with additional alterations that potentially contribute to disease progression (8, 9, 10, 11, 12, 13, 14, 15). This was illustrated in the comparison of ATC and WDTC tumors in the GATCI cohort, where ATC tumors recapitulated the characteristic features of WDTC (including the truncal MAPK-activating alterations as well as recurrent focal and arm-level CNAs such as 1q amplification and 22q deletion) while also harboring numerous recurrent SNVs and CNAs not commonly seen in well-differentiated tumors (8). Several of the mutations associated with the progression of the disease (e.g., mutations in *TP53* and members of the PI3K/AKT pathway) also exhibit a clear pattern of stepwise increase in prevalence as the tumors become more aggressive, being rare in PTC, more frequent in poorly differentiated thyroid cancer (PDTC), and most common in ATC (25). Furthermore, a co-occurring differentiated thyroid cancer (DTC, including WDTC and PDTC) component is observed in 20–50% of ATC tumors (12, 14, 15, 18), suggestive of a branching evolution during the course of disease progression (Fig. 1B). Subclonal reconstruction of the paired ATC and co-occurring DTC from nine patients in the GATCI cohort showed significant overlap between the alterations in these components in all cases and confirmed their shared evolutionary origin (8). The shared genomic alterations were particularly striking when evaluating CNAs, where the two components presented about 95% overlap (Fig. 1B) (8). Paired with mechanistic investigations in genetically engineered mouse models showing the progression of tumors to PDTC and ATC when MAPK-activating alterations are combined with additional alterations enriched in advanced tumors (22, 23, 24), these findings overall solidify the framework that the sequential acquisition of oncogenic alterations drives the advancement to ATC.

The subsequent sections highlight the frequent genomic alterations in ATC and examine their biological and clinical ramifications, starting with the tumorigenic MAPK-activating alterations and continuing with the aberrations enriched in the advanced disease. Given the modest sample size of most ATC genomics studies and the commonly low tumor purity of ATCs, which raises technical difficulties in the accurate estimation of the alteration rates, the median along with the interquartile range (IQR, expressed as Q1–Q3) of the alteration rates is reported for the relevant genes detailed throughout this review.

### MAPK pathway

**Figure 2:**
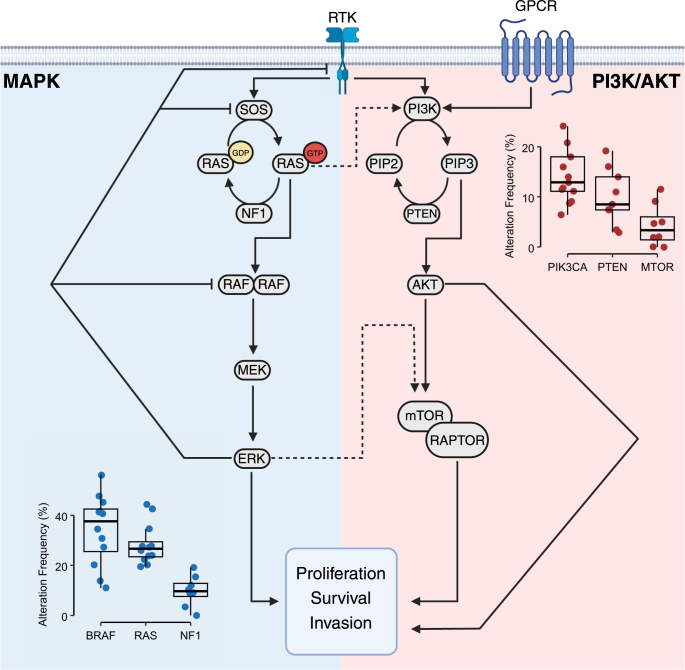
Key components of MAPK and PI3K/AKT pathways in ATC. The canonical MAPK pathway is initiated by the RTKs binding to their ligands. This binding induces dimerization and activation of the RTKs which, in turn, recruit the guanine nucleotide exchange factor (GEF) SOS and promote the exchange of GDP for GTP on RAS, thereby activating it. Subsequently, RAS induces the dimerization and activation of RAF proteins. This is followed by sequential phosphorylation and activation of MEK and ERK. ERK then mediates the downstream functions of the pathway by interacting with a multitude of effectors. ERK also participates in the negative regulation of MAPK signaling through direct interaction with the members of the pathway, as well as upregulation of negative regulators of MAPK signaling. PI3K is activated downstream of both RTKs and GPCRs. PI3K catalyzes the transfer of a phosphate group to phosphatidylinositol 4,5-bisphosphate (PIP2), converting it to phosphatidylinositol 3,4,5-trisphosphate (PIP3). PIP3 then induces the activation of AKT. Activation of AKT leads to derepression and activation of the mTOR complex 1 (mTORC1; presented as mTOR interacting with RAPTOR). AKT and mTORC1 are the primary nodes orchestrating the diverse functions of the PI3K/AKT pathway by interacting with numerous effectors. The rates of mutations in selected genes in ATC shown on the plots are derived from references (8, 9, 10, 11, 12, 13, 14, 15, 16, 17, 18, 19, 20).

The canonical MAPK pathway is a highly conserved kinase cascade that adjusts the molecular circuits within the cells in response to extracellular cues (Fig. 2). This pathway regulates diverse cellular processes, including proliferation and migration (26). MAPK signaling is frequently exploited during tumorigenesis, where its upregulated activity contributes to excessive growth rates, enhances the anti-apoptotic response, and increases the invasiveness of malignant cells (27). It is also thought to contribute to tumor growth by modulating the interaction of the tumor and immune system, at least partially through enhancing immune evasion by downregulating the major histocompatibility complex class 1 gene expression (28).

Genomic studies have shown that about 60–80% of ATC tumors harbor genomic alterations in the members of the MAPK pathway (8, 9, 10, 11, 12, 13, 14, 15, 16). These alterations mostly lead to constitutive activation of the MAPK pathway in thyroid follicular cells. In addition to enhancing the invasiveness and proliferation rates, elevated MAPK signaling in thyrocytes is associated with a regression in the tissue-specific cell state (29). This is likely due to the downregulation of thyroid differentiation genes as a consequence of ERK-induced disruption of the thyroid-stimulating hormone (TSH) signaling (30). Moreover, similar to melanoma, the activation of the MAPK pathway in thyrocytes is thought to enhance immune evasion (31) and contribute to an immunosuppressive microenvironment characterized by infiltration of pro-tumorigenic tumor-associated macrophages and myeloid-derived suppressor cells (32, 33, 34). Tumor-associated macrophages are, in particular, strongly implicated in the progression and aggressiveness of thyroid cancer, with ATC tumors exhibiting a distinctly heavy infiltration by these cells (34, 35, 36, 37). Although insufficient to fully sustain the progression to ATC in isolation (21, 38, 39), upregulated MAPK activity in thyroid follicular cells is a critical initiating step in ATC transformation (Fig. 1B).

Recurring mutually exclusive mutations in *BRAF* and RAS genes constitute the majority of the MAPK-activating alterations in ATC and are observed in 37.6% (IQR: 25.5–42.5%) and 26.6% (IQR: 23.4–29.4%) of cases (Fig. 2) (8, 9, 10, 11, 12, 13, 14, 15, 16, 17, 18, 19, 20). The most prevalent alteration in BRAF is the BRAF^V600E^ mutation: the conformational changes caused by the substitution of a valine residue with the negatively charged phosphomimetic glutamic acid in the activation loop of the protein render BRAF constitutively active and allow it to signal as a monomer (40, 41). In addition to having critical clinical implications (discussed later in this review), the monomeric signaling by BRAF^V600E^ has important biological consequences. In normal cells, the MAPK signal is relayed through RAS-induced RAF dimers (42), which are subject to negative feedback by ERK (Fig. 2). Activation of the signaling through RAF dimers is also the oncogenic mechanism of almost all other characterized MAPK-activating alterations, including alterations in *BRAF* that do not lead to the substitution of the V600 (41). However, the RAS-independent monomeric signaling by BRAF^V600E^ appears insensitive to the negative feedback by ERK and therefore can lead to a comparatively higher MAPK output, exhibiting a more potent transforming capacity (21, 38, 39, 43, 44, 45). Among the three RAS isoforms, mutations in *NRAS* are more prevalent than *HRAS* and *KRAS* in ATC and constitute 64.4% (IQR: 52.8–70.7%) of all RAS mutations (8, 9, 10, 11, 12, 13, 14, 15, 16, 17, 18, 19, 20). These mutations almost exclusively involve disruption of the GTPase activity of NRAS through alteration of codon Q61, locking NRAS in its active state.

Among the other notable alterations in the MAPK pathway are recurrent *NF1* alterations in 9.7% (IQR: 7.6–12.8%) of cases (8, 10, 11, 12, 13, 14, 15, 16) and rare alterations and fusions involving receptor tyrosine kinases (RTKs) (9, 10, 12, 18, 19). Neurofibromin, a GTPase-activating protein encoded by *NF1*, stimulates the GTP hydrolysis by RAS and its reversion to the GDP-bound inactive state (Fig. 2) (46). Loss of NF1 can therefore prolong the maintenance of the active state of RAS and heighten the MAPK output. Notably, alterations in *NF1* are enriched in ATC (8, 10), and, as shown in the GATCI cohort, copy number loss of *NF1* is frequent in these tumors (8). Interestingly, loss of neurofibromin is also associated with the disruption of adenylyl cyclase activity (47), which can potentially dysregulate TSH signaling, but whether this is through upregulated ERK activity or another mechanism is currently unknown. RTK fusions in ATC include RET fusions (12, 19), NTRK fusions (10, 12), and ALK fusions (18, 48, 49). The oncogenic activity of these fusions is generally through upregulation of the expression of RTKs, as well as the fusion partner-driven enhanced oligomerization leading to the constitutive activity of the kinase domains of the chimeric proteins independent of the extracellular signals (50).

As expected, the primary MAPK-activating alteration in DTC dictates the magnitude of MAPK upregulation, with tumors harboring BRAF^V600E^ displaying comparatively higher MAPK activity due to differential responsiveness to the negative feedback by ERK (9, 25, 51). Moreover, there is an inverse association between the intensity of the MAPK output and the differentiation state of the tissue in these tumors (9, 25, 51). Interestingly, these associations seem to be lost in ATC, where there is ubiquitously high MAPK activity and no apparent positive association between the intensity of the MAPK output and differentiation (9). Together, these suggest the contribution of additional mechanisms to the elevated MAPK signaling and dedifferentiation in ATC.

### PI3K/AKT pathway

PI3K/AKT is another pathway commonly co-opted during tumorigenesis and cancer progression (52). Operating alongside one another in mediating key processes such as proliferation, survival, and migration in response to extracellular signals (Fig. 2), the PI3K/AKT and MAPK pathways present substantial convergence on their downstream effectors and influence each other through a context-dependent crosstalk that remains incompletely understood (53). Beyond their convergence on the downstream functions and targets, two nodes in the MAPK pathway are known to positively regulate the PI3K/AKT pathway: active GTP-bound RAS can bind and cross-activate PI3K, and activated ERK functions similarly to AKT in activating the mTOR complex 1 (Fig. 2).

Alterations in the members of the PI3K/AKT pathway in thyroid cancer can co-occur with MAPK-activating alterations and are enriched in the aggressive forms of the disease, with about 20–40% of ATC cases harboring such alterations (8, 9, 10, 11, 12, 13, 14, 15). Upregulation of the PI3K/AKT pathway in ATC commonly occurs through activating mutations in *PIK3CA* (encoding the p110α catalytic subunit of PI3K; altered in 12.9% (IQR: 11.1–18%) of cases), *AKT1* (altered in 4% (IQR: 1–5.7%) of cases), and *MTOR* (altered in 3.4% (IQR: 1.4–6%) of cases) (8, 9, 10, 11, 12, 13, 14, 15, 16, 17, 19, 20). Loss-of-function (LOF) alterations in *PTEN* (altered in 8.5% (IQR: 7.4–14%) of cases), which encodes the primary inhibitor of the pathway, can also lead to elevated PI3K/AKT activity. In accordance with the close association of the two pathways, the alterations in the members of the PI3K/AKT pathway exhibit specific patterns of co-occurrence with MAPK-activating mutations present in tumors. Specifically, genomic aberrations of *PIK3CA* and *AKT1* tend to co-occur with *BRAF* mutations, while *PTEN* alterations mostly co-occur with *NF1* alterations in ATC (8, 9, 10, 11, 12, 13, 14, 15, 16, 17, 18, 19, 20). Importantly, functional studies in both human isogenic cell lines and genetically engineered mouse models have confirmed the association of the co-occurrence of *PIK3CA* and *BRAF^V600E^* mutations with thyrocyte dedifferentiation, aggressiveness, and progression to ATC (24, 54).

### Cell cycle and DNA damage response pathways

The most extensively characterized alteration in this category in ATC is the loss of p53. Along with the activation of TERT, alterations in *TP53* constitute the most prevalent alterations in ATC and are observed in 57% (IQR: 49.6–63.4%) of tumors (8, 9, 10, 11, 12, 13, 14, 15, 16, 17, 18, 19, 20). In stark contrast, *TP53* alterations are seen in less than 1% of well-differentiated papillary thyroid cancer cases (51), and while they present a higher rate of occurrence in advanced forms of DTC such as PDTC, they are uniquely prevalent in ATC (25). Extensive functional investigations have shown that the loss of p53 cooperates with MAPK-activating alterations to promote the development of ATC (22, 55, 56, 57, 58). Mechanistically, loss of p53 lifts constraints on the accumulation of genomic aberrations and promotes genomic instability, contributing to the higher SNV and CNA burdens in ATC compared to WDTC (Fig. 1A). This is also reflected by the association of the *TP53* mutation with the CNA subtypes identified in the GATCI cohort (8). Moreover, loss of p53 diminishes the restrictions on the intensity of MAPK output (30, 59) and can itself contribute to MAPK signaling (60), which may lead to the high levels of MAPK activity that do not exclusively depend on the primary MAPK-activating alteration in ATC. Altogether, somatic alterations of *TP53* are established drivers of the progression of malignant thyrocytes to ATC and are associated with many characteristic features of these aggressive tumors (22, 55, 56, 57, 58).

Alterations in numerous other genes involved in the cell cycle and DNA damage response have been observed in ATC (8, 9, 10, 11, 12, 13, 14, 15, 16, 17, 18, 19, 20). These include mutations in genes encoding members of the mismatch repair pathway (*MLH1*, *MSH2*, *MSH6*) and regulators of single-strand and double-strand DNA breaks (*ATM* and *ATR*). Widespread mutations and deletions of *BRCA1* and *BRCA2* were also highlighted in the GATCI cohort (8). In addition, alterations in *CDKN2A*, which encodes the central mediator of cell cycle arrest p16, are common in ATC, with the deletion of this gene representing one of the most frequent CNAs (it was observed in 42% of ATC cases in GATCI) (8, 10, 13). Overall, while there are limited functional investigations of these alterations in ATC, they may play oncogenic roles by contributing to genomic instability and the deregulation of the cell cycle (61, 62).

### TERT

Activation of TERT, the catalytic component of telomerase, is another highly prevalent alteration in ATC, with mutations in the *TERT* promoter seen in 50% of cases (IQR: 38.7–56%) (8, 9, 10, 11, 12, 13, 14, 15, 17, 18, 20, 63, 64, 65, 66). Alterations in *TERT* are implicated in the aggressiveness of thyroid cancer and are associated with worse patient outcomes, especially when they co-occur with alterations in *BRAF* or RAS genes (12, 65, 67, 68, 69). However, unlike *TP53*, alterations in *TERT* are also similarly prevalent in the advanced forms of DTC and are not specifically enriched in ATC (25). *TERT* promoter mutations include mutually exclusive c.-124C>T and c.-146C>T hotspot mutations, with the former comprising 90.2% (IQR: 87.5–92%) of cases (8, 10, 13, 14, 17, 18, 63, 64, 65). Notably, *TERT* promoter mutation is not the only mechanism through which thyroid tumors achieve TERT activation, with promoter hypermethylation and copy number gains representing alternative mechanisms (70, 71).

The canonical function of TERT is the maintenance of telomeres, and its reactivation confers replicative immortality to malignant cells (72). However, TERT is also implicated in various other telomere-independent cellular functions (73). While still not fully understood, the activity of TERT in thyroid cancer is thought to both allow malignant thyrocytes to escape replicative senescence (71) and contribute to cancer aggressiveness through extra-telomeric functions (23, 74). The activation of Tert in Braf^V600E^-mutant mouse models leads to the development of PDTC and ATC (23, 74). Tert activation in these models appears to induce higher MAPK and PI3K/AKT outputs and contribute to ribosome biogenesis and thyrocyte dedifferentiation (23, 74). The induction of MAPK output by Tert may explain the worse outcomes observed in patients when TERT activation is combined with BRAF or RAS alterations. Both hotspot mutations in the *TERT* promoter generate binding sites for ETS transcription factors (75), which are effectors downstream of the MAPK pathway. Consequently, the combination of activation of the MAPK pathway and *TERT* promoter mutations generates a feedback loop whereby the high MAPK output induces the expression of TERT, and active TERT enhances MAPK activity, leading to further dedifferentiation, replicative immortality, and more aggressive phenotypes.

### EIF1AX

*EIF1AX* encodes the EIF1A component of the 43S translation preinitiation complex (PIC), and its mutations deregulate the process of mRNA translation. *EIF1AX* mutations are observed in about 1% of well-differentiated papillary thyroid tumors and are mutually exclusive with other oncogenic drivers (51). In contrast, *EIF1AX* mutations are enriched in less differentiated, more aggressive forms of thyroid cancer, with about 8.5% (IQR: 2.2–13.7%) of ATC tumors harboring such mutations (8, 9, 11, 12, 13, 14, 16, 20). Moreover, genomic studies have consistently shown that *EIF1AX* mutations in ATC almost exclusively co-occur with RAS alterations (8, 9, 10, 11, 12, 13, 20), suggesting a cooperative association in disease progression. *EIF1AX* mutations in ATC occur either in the region encoding the N-terminal domain of the protein or at the A113 splice site that affects the C-terminal, with the latter appearing to be exclusive to thyroid cancer (8, 9, 10, 11, 12, 13, 20, 51). The oncogenic capacity of EIF1AX^A113splice^ has been demonstrated in genetically engineered mouse models where, concordant with the clinical manifestations, mutant EIF1AX in isolation may drive low-grade PTC, while the combination of alterations in RAS and EIF1AX can lead to less differentiated and more aggressive tumors (76). Functionally, the mutant EIF1AX increases global protein synthesis via an EIF2α-independent enhancement of the translation and transcription of ATF4, followed by the induction of the dephosphorylation of EIF2α through a negative feedback loop (76). Mutant EIF1AX and RAS also cooperate to stabilize c-MYC and increase the influx of amino acids, which in turn activate mTOR (76). Nevertheless, the convergence on c-MYC does not fully explain the particular association of *EIF1AX* mutations with the activation of RAS (as opposed to other MAPK-activating alterations), and additional mechanisms, such as the enhanced PI3K activity in RAS-mutant cells when EIF1AX is mutated (76), likely play a role in this context.

### Epigenetic regulators

Frequent alterations in genes encoding histone methyltransferases, histone acetyltransferases, members of the SWI/SNF chromatin remodeling complexes, and other epigenetic regulators are observed in ATC (8, 9, 10, 11, 12, 13, 14, 15, 16). These mutations are enriched in advanced thyroid cancer and may play oncogenic roles through mechanisms such as epigenetically mediated dysregulations of transcriptional programs and DNA damage response (77, 78). Studies investigating the epigenetic landscape of thyroid cancer have proposed several alterations in ATC, including global hypomethylation, hypermethylation of the promoters of *CDKN2A*, *PTEN*, and *TSHR*, as well as hypomethylation of the promoters of *MTOR*, *NOTCH1*, and *HIF1A* (79, 80, 81, 82). However, the functional consequences of the genomic alterations of epigenetic regulators in thyroid cancer are largely unexplored. The exceptions to this are alterations perturbing the activity of the SWI/SNF complexes, which have been shown to reduce chromatin accessibility and expression of lineage transcription factors, as well as other critical genes for the specialized function of thyrocytes (83). This is consistent with the characterized role of the SWI/SNF complexes in lineage specification (78). Moreover, the same study has shown that LOF of the members of the SWI/SNF complexes induced progression to PDTC and ATC in BRAF-mutant mice and abolished the redifferentiation effects of MAPK inhibition in these models (83).

### Genomic alterations with limited characterization

As discussed, mechanistic investigations have characterized the consequences of many of the prominent genetic alterations in ATC. Nonetheless, numerous alterations with potential roles in thyroid cancer tumorigenesis and progression remain uncharacterized, including an array of SNVs and CNAs enriched in ATC compared to DTC (8, 9, 10, 11, 12, 13, 16). Further research into the functional implications of these alterations could reveal novel oncogenic dynamics in thyroid cancer and may expose clinically useful therapeutic vulnerabilities of subsets of ATC tumors. For instance, recurrent mutations of *USH2A* and *LRP1* were found in both the GATCI cohort and a previous cohort of 22 ATC tumors characterized by whole-exome sequencing (8, 11). GATCI also strongly underscored the role of CNAs in ATC. The CNA subtypes identified in this study were associated with the clinical profiles of patients, and numerous recurrent CNAs with potential oncogenic roles were discovered. Indeed, frequent deletions of several tumor suppressors, such as *CDKN2A* and *BRCA2*, which were discussed above, were observed in cases with no known drivers in GATCI (8), suggesting their potential roles as drivers in a subset of cases. Furthermore, widespread losses of *CDKN2A* (which promotes cell cycle arrest through the inhibitory effects of its corresponding protein on CDK4 and CDK6) and *BRCA* genes provide the rationale for the preclinical evaluation of CDK4/6 inhibitors and PARP inhibitors in combination with other treatments as potential precision therapeutic interventions in patients harboring these alterations (84, 85).

## Advancements in the clinic: targeted treatments and immunotherapy

Significant strides have been made in redefining the historically dismal clinical management of ATC. In particular, the realization of the critical reliance of most ATC tumors on the upregulated MAPK pathway activity, coupled with the availability of small molecule inhibitors against the members of this pathway, raised excitement about the possibility of genotype-guided precision therapeutic interventions. Indeed, established genotype-matched treatments are currently available to several subgroups of ATC patients in the clinic: the combination of dabrafenib and trametinib (or vemurafenib and cobimetinib) for cases with the *BRAF^V600E^* mutation, larotrectinib or entrectinib for cases with NTRK fusions, and selpercatinib or pralsetinib for cases with RET fusions (86). Targetable ALK fusions are also rarely observed in ATC, providing another opportunity for therapeutic intervention (18, 48, 49). Given the capacity of the genomic alterations to inform the choice of treatment in the clinic, rapid molecular profiling of the tumors at the time of diagnosis through hotspot mutation testing and targeted next-generation sequencing panels is now strongly recommended (1). Yet still, over 60% of patients present with tumors that do not harbor these targetable alterations and thus have more limited treatment options (8, 9, 10, 11, 12, 13, 14, 15). Furthermore, the 6.7 months of progression-free survival among the ATC patients receiving the combination of dabrafenib and trametinib attests to the rapid development of resistance to this intervention, an occurrence that is almost universal in ATC (87). Thus, understanding the mechanisms of resistance and further development and enhancement of the treatment modalities available to patients suffering from ATC are active areas of investigation.

### Opportunities and challenges of MAPK inhibition in ATC

**Figure 3:**
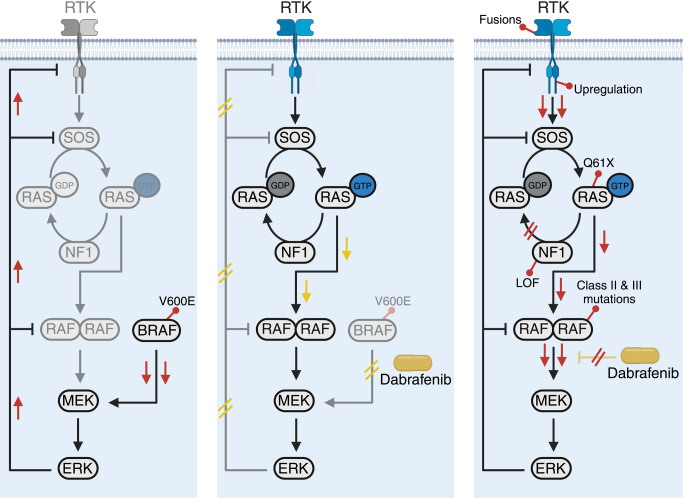
Response and resistance to first-generation RAF inhibitors. The first panel illustrates MAPK signaling in BRAF^V600E^-mutant cells. In these cells, monomeric signaling by BRAF^V600E^ leads to continuous activation of ERK and suppression of dimeric signaling through the negative feedback by ERK. The second panel shows the inhibition of the upregulated MAPK output by first-generation RAF inhibitors. First-generation RAF inhibitors (represented by dabrafenib) selectively inhibit the monomeric signaling by BRAF^V600E^, but they promote dimeric signaling by RAF through direct recruitment of RAF to RAS and stabilization of dimerization, as well as through relief of the feedback inhibition by ERK. These mechanisms might contribute to the development of adaptive resistance to first-generation RAF inhibitors. The third panel shows adaptive/acquired resistance to first-generation RAF inhibitors. Any mechanism that leads to dimeric signaling by RAF causes innate, adaptive, or acquired resistance to first-generation RAF inhibitors. These include RAS mutations, NF1 LOF, BRAF class II and III mutations (mutations other than alteration of the V600 that lead to either RAS-independent or RAS-dependent dimerization of RAF), and RTK fusions or upregulation.

The combination of dabrafenib and trametinib exhibits an objective response rate of about 60% in BRAF^V600E^-mutant ATC patients and is now part of the standard of care in this subgroup of patients, typically offered as the first line of therapy in the settings of locoregionally advanced unresectable tumors and metastatic disease (1, 87). Dabrafenib and vemurafenib are members of the first generation of RAF inhibitors and are widely explored as targeted treatments against BRAF^V600E^-mutant tumors (88). These ATP-competitive inhibitors have specific properties that confer a high therapeutic index to them and, at the same time, simplify the development of resistance against them (Fig. 3).

Notably, the binding of vemurafenib or dabrafenib to one of the protomers in a RAF dimer causes conformational changes that sterically impede the binding of an inhibitor to the second protomer (89). This means that, while these compounds restrict monomeric signaling by BRAF^V600E^, they do not effectively inhibit the MAPK signaling through RAF dimers. This selectivity broadens the therapeutic index of vemurafenib and dabrafenib (as they spare the MAPK signaling through RAF dimers in normal cells) but also renders them futile against malignant cells that signal through RAF dimers (e.g., NRAS-mutant cells).

Added to this is that first-generation RAF inhibitors stimulate RAF dimerization and can even paradoxically activate the MAPK pathway (90, 91). Two mechanisms are thought to contribute to the promotion of RAF dimers by first-generation RAF inhibitors. First, the direct interaction of these inhibitors with wild-type BRAF enhances the recruitment of the protein to RAS and facilitates dimerization (89, 92). This can lead to the paradoxical activation of the MAPK pathway in cells harboring active RAS and wild-type BRAF, which likely underlies some of the toxicities associated with these treatments, such as the development of cutaneous malignancies (88). Second, in BRAF^V600E^-mutant cells, the upregulation of the activity of ERK through monomeric signaling continually induces the suppression of dimeric signaling through negative feedback (Fig. 3). When treated with first-generation RAF inhibitors, suppression of the monomeric signaling leads to the relief of the negative feedback by ERK (43). This can trigger the reactivation of ERK through a rebound in dimeric RAF signaling, which is resistant to inhibition by first-generation RAF inhibitors (Fig. 3) (43).

Overall, these mechanisms highly restrict the efficacy and durability of current RAF inhibitors in the clinic. The lower efficacy of MAPK inhibition in ATC (and colorectal cancer) compared to melanoma is also likely, at least in part, due to the differential susceptibility of the different cell types to activation of dimeric signaling, as well as higher levels of dimeric signaling at baseline (89, 93, 94), although further investigation in this area is required. The addition of MEK inhibitors has been shown to reduce cutaneous side effects and improve efficacy in melanoma (95) and is also associated with enhanced outcomes in ATC (87, 96), most likely by counteracting paradoxical activation in normal cells and attenuating the MAPK activity rebound in malignant cells. As mentioned, it is indeed this combination of RAF and MEK inhibitors (dabrafenib and trametinib) that is now established as the standard of care in ATC and is recently FDA-approved tissue-agnostically (with the exception of colorectal cancer) in BRAF^V600E^-mutant tumors (97). Nevertheless, ATC patients almost invariably develop resistance to the current combinations of RAF and MEK inhibitors in the clinic, and while other potential mechanisms are also implicated in thyroid cancer (such as mutations in members of SWI/SNF or the PI3K/AKT pathway), reactivation of signaling through RAF dimers (for instance through mutations in RAS or NF1, enhanced RTK activity, or generation of self-dimerizing RAF isoforms; Fig. 3) is thought to be the primary mechanism of development of adaptive and acquired resistance to the current RAF inhibitors (98, 99, 100, 101). Accordingly, preclinical and clinical efforts exploring other types of RAF inhibitors are underway. These include investigations exploring type II RAF inhibitors such as naporafenib and belvarafenib, which are active against RAF dimers. These type II RAF inhibitors could potentially be used against tumors with initiating MAPK-activating alterations other than the *BRAF^V600E^*, as well as tumors with developed resistance against the current inhibitors through dimeric RAF signaling (102). Considering how these inhibitors also restrict MAPK signaling through RAF dimers in normal cells, they are predicted to have a narrower therapeutic index, although the sparing of ARAF, which appears to be a property of many compounds in this category (103, 104), might attenuate the treatment-related toxicity of these drugs. While the combination of type II RAF inhibitors and MEK inhibitors has been shown to be tolerated in multiple clinical trials in different types of cancer (105, 106, 107, 108), limited preclinical and clinical data regarding the tolerability and efficacy of these compounds in ATC are available, but ongoing efforts are rapidly closing the gap (109).

### The evolving frontier of immunotherapy in ATC

Finally, in contrast to the available data indicating an objective response rate of less than 10% for immune checkpoint blockade in DTC (110, 111), immune checkpoint inhibition in ATC appears more promising and is advancing in clinical trials. This differential sensitivity of ATC tumors to immunotherapy is not surprising, considering the profound differences in the microenvironment of ATC and DTC. Detailed characterization of the microenvironment of ATC is an active area of investigation. The results so far suggest that, compared to PTC, the microenvironment of ATC is enriched with specific fibroblast populations that support tumor growth, has a significantly higher proportion of immune cells, and exhibits an over-representation of immunosuppressive macrophages and lymphoid cells expressing high levels of exhaustion markers, a milieu that is speculated to be more amenable to modulation through immunotherapeutic interventions (37, 112, 113, 114, 115, 116, 117). An early clinical trial reported an objective response rate of 19% (29% among the PD-L1 positive cases) in 42 ATC patients treated with spartalizumab (118). This included three cases of complete response. Based on these results, the American Thyroid Association guidelines recommend that immune checkpoint blockade may be offered as first-line therapy (when other targetable alterations are not present) or at later stages of treatment (such as when resistance to the initial targeted therapy has emerged) for metastatic ATC with high PD-L1 expression, optimally as part of a clinical trial (1). Moving beyond monotherapy with immune checkpoint inhibitors, the objective response rate of dual checkpoint inhibition with durvalumab and tremelimumab in ATC patients in the DUTHY trial (*n* = 12) was 33.3% in the entire cohort and 50% in patients with PD-L1 positive tumors (119). Similarly, in a recent clinical trial assessing the efficacy of dual immune checkpoint blockade with nivolumab and ipilimumab in aggressive thyroid cancer, the clinical benefit rate in ATC patients (*n* = 10) was 50%, including three cases that experienced partial response and two cases of stable disease (120). With the establishment of targeted treatment with MAPK inhibitors in the ATC standard of care, assessment of the efficacy of the combination of MAPK inhibitors with immune checkpoint blockade represents an exciting new frontier in the clinical management of ATC. This is of particular interest, as, given the association of MAPK signaling with modulation of the interaction of the tumor and immune cells (32, 33), MAPK inhibition appears to lead to an incompletely understood remodeling of the immune microenvironment. Furthermore, the immunosuppressive microenvironment of the tumors is associated with resistance to MAPK inhibition (33, 121). These indicate that these two treatment modalities may act synergistically, but further research in this area is needed. The available data so far suggest superior patient outcomes when targeted inhibition of the MAPK pathway is combined with immunotherapy (15, 122), with a recent clinical trial reporting a remarkable median overall survival of 43 months in 18 BRAF^V600E^-mutant ATC patients receiving atezolizumab in addition to vemurafenib and cobimetinib (7). Overall, these results indicate the effectiveness of immunotherapy in ATC, warranting further evaluation of the incorporation of immune checkpoint blockade, alone or in combination with targeted treatments, in the ATC standard of care.

## Conclusion

The last decade witnessed significant progress in both our understanding of the molecular basis of ATC and the clinical management of the disease. Despite the aggressive characteristics of ATC tumors, the genomic landscape of ATC is only moderately complex (8) and is marked by numerous highly recurring features (8, 9, 10, 11, 12, 13, 14, 15, 16, 17, 18, 19, 20). Furthermore, the close evolutionary association of ATC with WDTC is now well-substantiated, and many of the most prominent alterations implicated in the progression of well-differentiated tumors to ATC are characterized through functional investigations (8, 22, 23, 24). Yet, numerous alterations with clear enrichments in the advanced disease remain largely understudied, and while efforts such as GATCI have made substantial progress in closing the gap, additional oncogenic alterations likely remain unidentified. Specifically, the contributions of many genomic features such as structural variants, germline variants, and mitochondrial DNA alterations to the development, intratumoral heterogeneity, and treatment resistance in ATC are underexplored. Further investigations in these areas may offer new insights into the molecular foundations of ATC and reveal novel avenues for therapeutic interventions. While the incorporation of genotype-guided inhibition of the MAPK pathway into the standard of care for ATC has led to remarkable improvements in patient outcomes (87), their applicability to only a subset of patients and the almost universal evolution of resistance to these interventions pose great challenges that should be addressed through multidisciplinary efforts. Nonetheless, novel classes of small molecule inhibitors against the components of the MAPK pathway are under active investigation, and the combination of immunotherapy with inhibitors of the MAPK pathway appears promising (7). As the gaps in our understanding of ATC are bridged and innovative treatments emerge, the clinical landscape of ATC is rapidly turning from a grim history of dismal patient outcomes to a hopeful future of precision medicine and immunotherapy.

## Declaration of interest

The authors declare that there is no conflict of interest that could be perceived as prejudicing the impartiality of the work reported.

## Funding

This work was supported by the Canadian Institute of Health Research grant MOP 487005.
